# Mitral Valve Nonbacterial Thrombotic Endocarditis Associated With Antiphospholipid Syndrome in a Male Patient: A Comprehensive Case Report

**DOI:** 10.1155/carm/6425550

**Published:** 2026-02-27

**Authors:** Afshin Amirpour, Niloufar Razavi, Mahmoud Saeidi, Anahita Amirpour, Reyhaneh Zavar, Mohammadsadegh Sahebzade

**Affiliations:** ^1^ Department of Cardiology, Cardiovascular Research Institute, Isfahan University of Medical Sciences, Isfahan, Iran, mui.ac.ir; ^2^ Department of Cardiac Surgery, Isfahan University of Medical Sciences, Isfahan, Iran, mui.ac.ir; ^3^ Department of Rheumatology, Shahid Beheshti University of Medical Sciences, Tehran, Iran, sbmu.ac.ir

**Keywords:** antiphospholipid syndrome, cardiac neoplasm, case reports, marantic endocarditis, nonbacterial thrombotic endocarditis

## Abstract

Nonbacterial thrombotic endocarditis (NBTE) is a rare cardiac disease with a nonspecific presentation associated with hypercoagulable states including malignancy and autoimmune disorders, such as antiphospholipid syndrome (APS) and systematic lupus erythematosus. A 34‐year‐old male with a history of pulmonary thromboembolism presented to the hospital complaining of chest pain as an initial symptom. Transthoracic echocardiography revealed the presence of a mass‐like lesion adhering to the anterior leaflet of the mitral valve. Once infective endocarditis was excluded, further imaging studies suggested that the cardiac neoplasm was the primary cause of the patient’s symptoms, and the patient underwent surgical excision of mass along with mitral valve replacement. Microscopic analysis of the removed mitral valve mass indicated the presence of fibrotic tissue with thick collagen bundles and no inflammatory infiltrates, supporting the diagnosis of NBTE. Investigations for underlying conditions demonstrated persistent elevated titers of anti–β2 glycoprotein I IgG, supporting a diagnosis of APS. The patient was started on warfarin therapy with a closely monitored International Normalized Ratio (INR). After 6 months of follow‐up, the patient reported no complications. This case highlights the importance of considering NBTE as a potential differential diagnosis in patients with intracardiac mass and inspecting associated conditions such as APS, as well as challenges encountered in diagnosis and management.

## 1. Introduction

Antiphospholipid syndrome (APS) is an autoimmune condition associated with antiphospholipid antibodies, leading to thrombosis in major organ systems [1]. Clinical manifestations of APS include arterial, venous, and microvascular thrombosis; obstetric complications; cardiac valve involvement; and hematologic disorders [2]. For APS diagnosis, in addition to a clinical manifestation of thrombosis, laboratory evidence of persistent antiphospholipid antibodies including anticardiolipin (aCL), anti–β2 glycoprotein I (aβ2GPI), or lupus anticoagulant (LAC) is required [3].

Nonbacterial thrombotic endocarditis (NBTE), also known as marantic or Libman–Sacks endocarditis, is characterized by developing sterile thrombotic vegetation(s) on the cardiac valve structure and posing a risk for embolic stroke [4]. NBTE is often linked to hypercoagulable states such as malignancies and autoimmune disorders including APS and systemic lupus erythematosus (SLE) [5]. Diagnosing NBTE can be challenging and necessitates a high level of clinical suspicion. It is crucial to exclude infective endocarditis (IE) prior to diagnosing NBTE [6]. Transthoracic echocardiography (TTE) is the primary imaging modality, while transesophageal echocardiography (TEE) has been found to manifest higher sensitivity and specificity in the detection of NBTE [6]. Nevertheless, a definite diagnosis can be made by the presence of fibrin and platelets on surgical specimens [7]. Standard treatment for NBTE is uncertain, but it should focus on reducing embolic events and addressing valvular dysfunction to prevent cardiac function impairment, as well as managing underlying conditions [3, 4].

Since APS is predominantly diagnosed in females and NBTE is a rare etiology of intracardiac mass [8], the concurrent presence of APS and NBTE in men is likely to be exceedingly uncommon. This case report presents a 34‐year‐old male with a history of pulmonary thromboembolism diagnosed with mitral valve NBTE associated with APS.

## 2. Case Presentation

A 34‐year‐old man presented to the emergency department with retrosternal chest pain for one hour, rated as 8/10 in intensity, unrelated to exertion, and unrelieved by sublingual nitroglycerin. The pain was stable and not accompanied by dyspnea, orthopnea, paroxysmal nocturnal dyspnea, nausea, vomiting, dizziness, diaphoresis, or syncope. His medical history included pulmonary embolism (PE) 8 years earlier following a motor vehicle accident. He was taking 20 mg of rivaroxaban daily. His father had experienced myocardial infarction at age 46.

**FIGURE 1:**
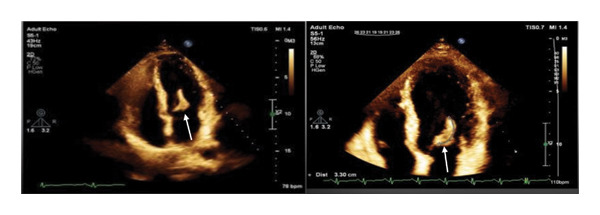
Apical four‐chamber view of transthoracic echocardiography. White arrows indicate an elongated, large, hypermobile mass with myocardial density measuring 44 by 11 mm, attached to the atrial side of the A3 scallop.

On admission, the patient was alert and oriented; vital signs were stable (blood pressure 130/80 mmHg, heart rate 84/min, respiratory rate 16/min, temperature 37°C), and cardiovascular, pulmonary, and abdominal examinations were unremarkable. The initial electrocardiogram (ECG) revealed normal sinus rhythm with ST‐segment depression in I and AVL leads without dynamic changes in the subsequent serial ECGs. TTE indicated a large, mobile mass attached to the atrial side of the anterior mitral leaflet with preserved ventricular function, raising suspicion for infective IE, for which empirical antibiotic therapy (ampicillin‐sulbactam and vancomycin) was initiated (Figure 1).

**FIGURE 2:**
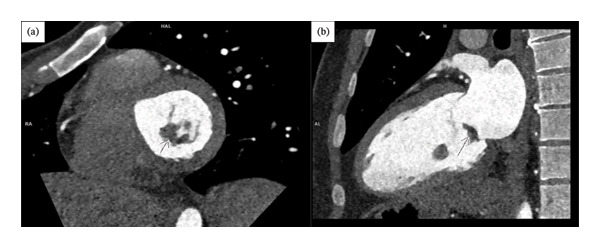
CTA showed a hypodense, mass‐like lesion on the atrial side of the anterior mitral valve leaflet (arrows). ((a) Short axis view at the level of mitral valve and (b) Sagittal view, CTA: computed tomography angiography).

**FIGURE 3:**
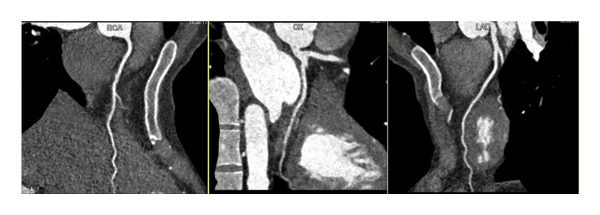
CCTA showed no significant coronary artery disease. (RCA: right coronary artery, LAD: left anterior descending artery, CX: circumflex artery, CCTA: coronary computed tomography angiography).

TEE demonstrated hypokinesia of the basal, mid‐inferior, and mid‐posterior myocardial segments and confirmed a hypermobile, broad‐based mass measuring 44 × 11 mm attached to the A3 scallop of the anterior mitral leaflet [9]. Laboratory evaluation demonstrated elevated serum troponin (137 ng/L), but blood cultures and inflammatory markers were negative (Table 1). Subsequent coronary and pulmonary computed tomography angiography (CTA), along with abdominal ultrasonography, confirmed the mitral valve mass lesion without evidence of coronary artery disease, PE, and malignancy (Figures 2 and 3). Given the clinical, laboratory, and imaging findings, the probability of IE was considered low, and a tumoral process was established as the leading diagnosis. Next, surgical mitral valve replacement (MVR) was planned in conjugation with continued antibiotic therapy.

**TABLE 1:** Laboratory investigations at the time of admission.

Lab	Value	Reference
WBC (10^3^/*µ*L)	7.9	3.5–11
Hb (g/dL)	15.5	13.2–18.5
PLT (10^3^/*µ*L)	77	130–450
ESR (mm/h)	21	0–22
CRP (mg/L)	2.8	0–10
Urea (mg/dL)	10.6	9.3–25
Cr (mg/dL)	1.4	0.7–1.4
PTT (s)	64	25–42
PT (s)	14.7	12–13.3
INR	1.15	2‐3
Blood culture	Negative	Negative
Troponin	Positive	Negative

*Note:* The table presents the relevant laboratory data collected at the time of admission.

Abbreviations: Cr, creatinine; CRP, C‐reactive protein; ESR, erythrocyte sedimentation rate; Hb, hemoglobin; INR, International Normalized Ratio; PLT, platelet; PT, prothrombin time; PTT, partial thromboplastin time; WBC, white blood cell count.

**FIGURE 4:**
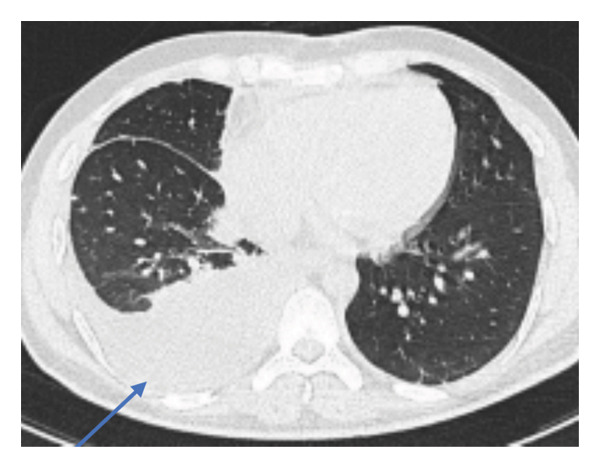
An axial view of HRCT demonstrated moderate right pleural effusion (blue arrow). (HRCT: high‐resolution computed tomography).

On the ninth day of the hospital stay, the patient underwent MVR with a mechanical prosthesis (Carbomedics Orbis 27), whereby a pathology specimen was sent. Postoperatively, warfarin was added to the treatment regimen, and the International Normalized Ratio (INR) was closely monitored. He remained afebrile for 8 days, after which he developed a fever and elevated C‐reactive protein, along with moderate pericardial and right‐sided pleural effusions on imaging. High‐resolution computed tomography confirmed the pleural effusion, which was treated with thoracentesis; cytological examination was negative for malignancy (Figure 4).

Histopathological examination of the excised valve tissue exhibited fibrotic thickening with dense collagen bundles and no inflammatory infiltrate or microorganisms; periodic acid–Schiff staining was negative. A second pathologist confirmed these findings, supporting the diagnosis of NBTE. Antibiotics were discontinued, and a comprehensive evaluation for underlying conditions was performed. Screening for malignancy, SLE, rheumatoid arthritis, and coagulation disorders was negative. Nevertheless, serological testing indicated elevated aβ2GPI IgG (92.30 U/mL), which remained positive at high titers upon repeat testing after 12 weeks (118 U/mL). The rheumatology team confirmed a diagnosis of APS (Table 2).

**TABLE 2:** Serological Findings associated with NBTE.

Lab	Value	Reference
Anti β2GPI IgM (U/mL)	0.80	< 10
Anti β2GPI IgG (U/mL)	118	**< 12**
Anti‐phosphatidylserine antibodies IgM (U/mL)	13.59	< 12
Anti‐phosphatidylserine antibodies IgG (U/mL)	74.22	**< 12**
ACA IgM (U/mL)	0.44	< 12
ACA IgG (U/mL)	8.41	< 12
Anti‐dsDNA antibodies	< 1:10	< 1:10
Anti‐Ro/SSA antibodies (U/mL)	4.55	< 12
RF (U/mL)	8.8	< 20
Brucellosis IgM	1.87	< 9
Brucellosis IgG	0.39	< 9
Wright agglutination test (Titer)	Negative	Negative
Combs Wright (Titer)	Negative	Negative
2ME (Titer)	Negative	Negative

*Note:* This table presents relevant laboratory data collected to investigate conditions associated with NBTE.

Abbreviations: β2GPI, β2glycoprotein I; 2ME, 2‐mercapto ethanol; ACA, anticardiolipin antibody; Anti‐ds DNA, antidouble‐stranded deoxyribonucleic acid antibodies; Anti‐SSA, anti‐Sjögren’s‐syndrome‐related antigen A; NBTE, nonbacterial thrombotic endocarditis; RF, rheumatoid factor.

The patient was maintained on long‐term warfarin anticoagulation (target INR 2–3), and at six‐month follow‐up, he remained free of thromboembolic or hemorrhagic complications.

## 3. Discussion

NBTE is a rare cardiac condition characterized by fibrin and platelet aggregation on the cardiac valve without evidence of an inflammatory response or bacteremia in the context of a hypercoagulable state [10]. The exact pathophysiology of NBTE is unclear; however, it is believed that endothelial injury caused by inflammatory markers, hypoxia, or local turbulence of blood flow in hypercoagulable patients results in the deposition of aggregated platelets and fibrin on cardiac structures [4].

There are no pathognomonic signs and symptoms for NBTE, and patients are usually asymptomatic until embolization occurs, with up to 50% of patients with NBTE presenting with embolic phenomena [7]. Clinical features suggestive of associated underlying conditions may be present, including weight loss, fever, concomitant paraneoplastic syndromes of malignancy, as well as clinical signs of autoimmune diseases such as skin rashes, oral and genital aphthae, arthralgia and arthritis, Raynaud’s phenomenon, pleural or pericardial effusions, thrombosis, and a history of recurrent miscarriages [6]. In the current admission, our patient experienced chest pain, which is not a common symptom based on the previous study by Venepally et al. [11], in which chest pain as an initial symptom was observed in only 11.7% (19 out of 163) of patients with NBTE.

Echocardiography is the initial method employed to evaluate patients with cardiac symptoms given its wide availability, excellent temporal‐spatial resolution, and ability to ascertain hemodynamic impacts. The differential diagnoses for cardiac masses include IE, thrombus, scar, inflammatory lesion, neoplasm, and foreign body [12]. IE should be excluded in patients with cardiac vegetation using modified Duke criteria. If the initial workup does not identify an infective pathogen, culture‐negative endocarditis should also be considered [13].

Cardiac neoplasms are categorized into primary and secondary (metastatic) forms. Primary tumors are rare, with an estimated prevalence of about 0.001% to 0.3% in autopsies [14]. The most common benign primary cardiac tumors are cardiac myxomas, followed by lipomas and fibroelastoma [15]. There are various imaging modalities such as echocardiography, cardiac magnetic resonance imaging (CMR), cardiac CT, and positive emission tomography (PET‐CT/MRI) which can be useful in characterizing masses and limiting different diagnoses. When imaging is not sufficient, histology of intracardiac masses is required for definitive diagnosis [12].

To the best of our knowledge, there is only one report that has indicated a connection between APS and NBTE presenting as a myxoma‐like mass in a male patient. In a report by Yordan‐Lopez et al. [16], NBTE was misdiagnosed as cardiac neoplasm in a male patient owing to similar imaging characteristics. In this study, the patient was older and presented with symptoms of heart failure as well as persistent right calf pain. He underwent surgical intervention because of worsening tricuspid valve regurgitation. In our study, the cardiac mass caused mild to moderate mitral valve regurgitation, and to definitively manage and diagnose the condition, the patient underwent surgical excision along with MVR.

Despite advances in imaging and laboratory investigations, there are no specific criteria for diagnosing NBTE, with postmortem or histological examination remaining as the gold standard for diagnosing NBTE [7]. The presence of fibrin and platelets along with the absence of microorganisms and inflammatory cells are the hallmarks of NBTE in pathology specimens [4]. Similar to our case, the diagnosis of NBTE was confirmed after surgical removal of the mass and pathologic examination in most previous similar case reports [16–19].

It is important to identify underlying inflammatory processes when NBTE is diagnosed in patients with no significant medical history [4, 6]. The work‐up should include age‐appropriate cancer screening and blood testing for antinuclear antibodies profile, rheumatoid factor, protein C and S, and factor V Leiden mutations. Additional serological testing for APS should be carried out, including LAC, IgG, and/or IgM aCL, and aβ2GPI. Further, CT of the chest, abdomen, and pelvis may be obtained to look for hematological and solid malignancies [4].

The 2023 ACR/EULAR classification criteria for APS employ a weighted, points‐based structure and begin with an entry requirement of at least one positive antiphospholipid antibody test (LAC, aCL, or aβ2GPI of the IgG or IgM isotype) within three years of a clinical event compatible with APS. Once this entry criterion is fulfilled, patients receive weighted scores across several clinical domains, including macrovascular venous or arterial thrombosis, microvascular involvement, obstetric manifestations related to placental insufficiency, cardiac valve abnormalities, and thrombocytopenia. Laboratory domains assign additional points based on the persistence of antibody positivity, moderate‐to‐high titers, and the presence of multiple antiphospholipid antibody types. APS classification requires a minimum of 3 clinical points and 3 laboratory points. Compared with the 2006 Sapporo criteria, the new system provides markedly higher specificity and incorporates nonclassical features such as microvascular and valvular involvement, although sensitivity may be reduced in certain subgroups, particularly those with isolated obstetric manifestations [20, 21].

According to the 2023 ACR/EULAR criteria, our patient satisfies both laboratory and clinical thresholds. He demonstrated cardiac valve involvement in the form of sterile vegetations consistent with NBTE, yielding 4 clinical points, and persistent high‐titer aβ2GPI IgG positivity, contributing 5 laboratory points. With a total score exceeding the threshold of ≥ 3 clinical and ≥ 3 laboratory points, the case meets the classification criteria for APS.

The treatment of NBTE typically involves managing the underlying autoimmune conditions, including malignancy, APS, and SLE [22]. Vitamin K antagonists such as warfarin are recommended for treating thrombotic APS [23]. The target INR should be established and closely monitored to ensure the efficacy of the treatment [24]. Evidence suggests that direct oral anticoagulants (DOACs) may not be suitable for APS, particularly in patients with a triple‐positive antibody profile [25]. Immunomodulatory agents such as hydroxychloroquine, rituximab, and vitamin D have proven benefits as adjuncts to standard anticoagulation therapy owing to the immune‐mediated nature of APS [26]. In our case, when the diagnosis of NBTE and APS was confirmed, anticoagulation therapy with warfarin was administered along with close INR monitoring.

## 4. Conclusion

NBTE is a rare condition, often undiagnosed, and is commonly associated with autoimmune disorders such as APS. Particularly in the early stages, differentiating NBTE from other intracardiac mass diseases including cardiac neoplasms and IE may be challenging to diagnose, which often results in a delayed diagnosis or postmortem discovery. Hence, in the case of suspected or confirmed NBTE, immunological workups are necessary to examine underlying conditions.

**Nomenclature**
NBTE: Nonbacterial thrombotic endocarditis
APS: Antiphospholipid syndrome
SLE: Systematic lupus erythematosus
IE: Infective endocarditis
TTE: Transthoracic echocardiography
TEE: Transesophageal echocardiography
MVR: Mitral valve replacement

## Funding

No funding was received for this manuscript.

## Consent

Written informed consent was obtained from the patient for publication of this manuscript and any accompanying images.

## Conflicts of Interest

The authors declare no conflicts of interest.

## Data Availability

The data that support the findings of this study are available from the corresponding author upon reasonable request.
